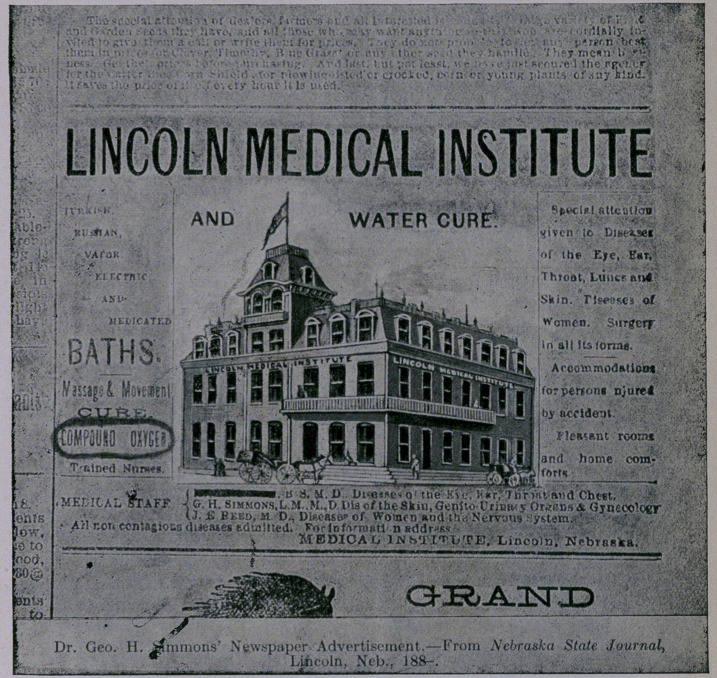# The Dictator of Medical Ethics

**Published:** 1909-12

**Authors:** 


### THE DICTATOR OF MEDICAL ETHICS.

Dr. Geo. H. Simmons, Editor *Journal A. M. A.*, and Secretary of the Association, when discovered (in the eighties) was running this cut in the Nebraska newspapers. It represented his (Homeopathic) Medical Institute—Water Cure and Compound Oxygen Establishment, at Lincoln, Neb. When asked about it by Lydston he said he had forgotten it.

## Figures and Tables

**Figure f1:**